# Joint learning sample similarity and correlation representation for cancer survival prediction

**DOI:** 10.1186/s12859-022-05110-1

**Published:** 2022-12-19

**Authors:** Yaru Hao, Xiao-Yuan Jing, Qixing Sun

**Affiliations:** 1 School of Computer Science, Wuhan University, Wuhan, China; 2 Guangdong Provincial Key Laboratory of Petrochemical Equipment Fault Diagnosis and School of Computer, Guangdong University of Petrochemical Technology, Maoming, China; 3 State Key Laboratory for Novel Software Technology, Nanjing University, Nanjing, China

**Keywords:** Cancer survival prediction, Feature information, Structure information, Correlation representation, Similarity matrix

## Abstract

**Background:**

As a highly aggressive disease, cancer has been becoming the leading death cause around the world. Accurate prediction of the survival expectancy for cancer patients is significant, which can help clinicians make appropriate therapeutic schemes. With the high-throughput sequencing technology becoming more and more cost-effective, integrating multi-type genome-wide data has been a promising method in cancer survival prediction. Based on these genomic data, some data-integration methods for cancer survival prediction have been proposed. However, existing methods fail to simultaneously utilize feature information and structure information of multi-type genome-wide data.

**Results:**

We propose a Multi-type Data Joint Learning (MDJL) approach based on multi-type genome-wide data, which comprehensively exploits feature information and structure information. Specifically, MDJL exploits correlation representations between any two data types by cross-correlation calculation for learning discriminant features. Moreover, based on the learned multiple correlation representations, MDJL constructs sample similarity matrices for capturing global and local structures across different data types. With the learned discriminant representation matrix and fused similarity matrix, MDJL constructs graph convolutional network with Cox loss for survival prediction.

**Conclusions:**

Experimental results demonstrate that our approach substantially outperforms established integrative methods and is effective for cancer survival prediction.

## Introduction

Cancer has been becoming the leading death cause all over the world, which seriously affects human health and living quality [1, 2]. In addition, the mortality rates increase year by year [3–5]. Prognosis prediction can aid physicians significantly in making decisions about care and treatment of cancer patients [6, 7]. Prognosis prediction usually can be described as a censored survival analysis problem, which predicts whether and when a death will occur within a given time period [8, 9]. In the past few decade, many survival prediction methods have been proposed, such as standard Cox regression and its extensions [10], tree-based ensemble methods, random survival forests [11], and so on.

Historically, cancer survival prediction works mainly based on histopathological descriptors and low-dimensional clinical data, such as sex, age at diagnosis, cancer grade detail, body fat rate and other clinical features [12–14]. However, clinical practice has found that genomic data tends to contain more molecular biomarkers associated with cancer and thereby can describe the cancer more comprehensively [15, 16]. Meanwhile, with the advance of Human Genome Project, high-throughput sequencing technology becomes cost-effective, which makes it progressively easier to achieve multiple and diverse genome-scale data sets to address clinical and biological questions [17]. In general terms, the above multi-type data describing the same cancer can be regarded as multimodal data. Specifically, multimodal data has two basic characteristics [18–20]. On the one hand, they share the common information both in feature level and structure level. On the other hand, each modality has its own specific information both in feature level and structure level. Compared with single genetic data type, multiple genome-scale data sets can capture more comprehensive information for cancer. Therefore, it is essential and feasible to develop new data-integration algorithms especially for utilizing multi-type high-dimensionality genomic data to capture comprehensive information for cancer.

### Motivation

During the past several years, many researchers have been devoted to construct data-integration methods based on binary classification models for cancer survival prediction. In this technology, cancer patients are usually classified to the short or long survival group according to a predefined threshold (e.g., 3 years). For example, Zhang et al. [21] presented a multiple kernel machine learning method combined with min-redundancy max-relevance (mRMR) feature selection algorithm to predict 2-year survival rate of glioblastoma multiforme patients. Zhao et al. [22] studied various prediction methods including ensemble models (Gradient Boosting and Random Forest), support vector machine and artificial neural networks to predict 5-year survival rate of breast cancer by fusing gene expression data, clinical data and pathological images. Unfortunately, this technology reduces the survival analysis to a classification problem, which is counter-practical and far less useful than the estimation of survival times. Another mainstream technology for survival prediction is survival risk regression based methods, such as Cox proportional hazards (Cox-PH) model [23, 24]. Different from binary classification methods, this technology focuses on whether a patient survives at a certain time point rather than when the patient dies, which can handle both uncensored and censored samples. Therefore, patients who survive at a certain time point can be used in modelling patient survivals [25].

Although existing works have promoted the development of data-integration methods in cancer survival prediction, there are two limitations to develop this technology: *(i) simultaneously utilizing structure information and feature information, specifically for small scale dataset; (ii) fully utilizing multi-type data for learning effective discriminant features. Here, structure information points to the information of data distribution within data types. Feature information refers to the information contained in the data (such as genes) within a sample. Discriminant features refer to the features learned from original data (such as gene sequences) by utilizing feature learning algorithms, which is useful to separate the samples with different survival time* [26]. Existing data-integration methods for cancer survival prediction have yet to address all of these limitations together. In addition, with excellent feature learning ability, the neural network extension of the Cox model has proved its better performance than traditional Cox-PH models in survival prediction, especially for high-throughput sequencing data. Hence, we intend to apply it to our work. In addition, we introduce similarity matrix to exploit structure information, which can access structural information hidden in multi-type data.

Inspired by the above analysis, we intend to design a Multi-type Data Joint Learning (MDJL) approach to obtain a reliable similarity matrix for exploiting structure information and an effective discriminant feature representation for exploiting feature information. In our proposed MDJL, (a) structure information and feature information can be simultaneously utilized; (b) the discriminant feature representations are exploited by learning correlation representations between any two data types, which can ensure the diversity and provide complementary information; (c) the constructed similarity matrices can explore useful structure information even from a small-scale samples.

### Contribution

The main contributions of our approach lie in three aspects:

1. Different from existing survival prediction methods, we present a Multi-type genome-wide Data Joint Learning (MDJL) approach for cancer survival prediction, which achieves both a fused similarity matrix and an integrated discriminant feature representation for simultaneously utilizing structure information and feature information.
2. MDJL exploits correlation representations between any two data types by cross-correlation calculation for learning discriminant features. Moreover, based on the learned correlation representations, MDJL constructs sample similarity matrices for capturing global and local structures across different data types. With the learned discriminant representations and similarity matrices, MDJL constructs graph convolutional network with Cox loss for survival prediction.
3. We conduct a number of experiments on four public cancer datasets. Experimental results show that our approach can achieve higher prediction performance than competing methods. Further investigation not only demonstrate the effectiveness of each component for MDJL, i.e., correlation representations extraction component and similarity matrices construction component, but also indicate the robustness.

### Organization

The rest of this paper is organized as follows: Sect. Motivation reviews related cancer survival prediction works. The proposed approach and detailed algorithm are introduced in Sect. Contribution. Section Organization talks about the experimental results. Section Related works conducts further experiments to investigate our approach. Section Binary classification based survival prediction works concludes this paper.

## Related works

### Binary classification based survival prediction works

In the past few decades, a variety of binary classification based multimodal learning methods for survival prediction have been proposed. In general terms, a modality refers to a kind of data type. These methods mainly focus on learning fused representation from multiple data sources, such as clinical data, histopathological images markers and genomic data [27–31]. With multiple types of data, some data-integration strategies such as joint-based strategy [32, 33] and alignment-based strategy [34–36] have been presented. Joint-based methods utilize multi-type data mainly by concatenating multi-type data into one unified feature matrix. For example, Sun et al. [37] presented a triple model DNN to respectively learn feature representations from gene expression, copy number alteration and clinical data, and then concatenated the learned multiple representations into one unified matrix. To explore the inherent relation between samples and multi-type genomic data, Gao et al. [38] constructed bipartite graphs between patients and gene expression, copy number alteration. Khademi et al. [39] integrated microarray data and clinical data through the probabilistic graph model for prognosis of breast cancer. Methods based on alignment strategy utilize multiple types of data by maximizing the common information across different data types. For example, Wang et al. [40] designed a cluster-boosted multi-task learning approach to exploit the common information across different data types for survival analysis. Although these methods have promoted the development of multimodal cancer survival analysis, they are limited to binary classification problem and are counter-practical.

### Survival risk regression based survival prediction works

Different from binary classification methods, the survival risk regression methods aim to calculate a risk score for each patient, typically with the Cox-PH model and its extensions [41–43]. For example, to predict an individual survival time, Baek et al. [44] achieved this by integrating hazard network and a distribution function network. Wang et al. [45] proposed a reweighted Lasso-Cox model for cancer survival prediction, which improves the generalization ability of the model by weighing the topologically important genes based on random walk. Considering there are correlations between multi-type genomic data, Bichindaritz et al. [46] presented an adaptive multi-task learning approach for breast cancer survival prediction, which add an auxiliary ordinal loss to the Cox model.

Recently, with the excellent data representation ability and high learning ability, a variety of deep neural networks extension of the Cox-PH model has been proposed [47–50]. For example, instead of learning linear relationship in the Cox-PH model, both DeepSurv [51] and Cox-nnet [52] introduce neural networks to learn nonlinear feature representation. To fully utilize multi-omics data, Tong et al. [53] designed a concatenation autoencoder to concatenate the learned multiple hidden representations from each data type. In addition, to achieve the consensus representation across multi-omics data, they designed a cross-modality autoencoder to maximize the agreement across modalities. Cheerla et al. [54] presented an unsupervised encoder extension of the Cox model to integrate multi-type data into one single feature matrix, which introduces similarity loss to force four data sources align the common information. To eliminate the estimation bias in processing such datasets with a large number of censored samples, Zhang et al. [55] introduced Bayesian Perturbation to approximate the prior knowledge of censored samples to optimize the training process of model. To address the limitation that deep networks tend to fall into over-fitting with small sample size high feature dimension, Qiu et al. [56] present a meta-learning approach based on neural networks for cancer survival prediction. In addition, Kvamme et al. [57] imposed $L_{1}$ and $L_{2}$ regulation terms on the network parameters to reduce the over-fitting problem. However, these methods mainly exploit feature information but fail to exploit useful structure information.

### Similarity matrices construction works

Similarity matrix construction has been widely used in multi-view clustering tasks. Usually, existing methods tend to construct similarity matrix for each data types, based on which they learn a shared similarity matrix of all data types. For example, Zhan et al. [58] learned the consensus similarity graph by minimizing disagreement between different views with a disagreement cost function. To address the limitation that incomplete multi-view clustering fails to exploit hidden information of missing views and handle the information imbalance across different views, Wen et al. [59] designed adaptive weights to balance the importance of different views. Wang et al. [60] designed a multi-view subspace clustering approach, which adopts the Hilbert-Schmidt Independence Criterion to enforce the similarity of similarity matrix have maximum dependence. Chen et al. [61] designed a nonlinear method for multi-view clustering, which jointly learn kernel representation matrix and similarity matrix. Zhang et al. [62] presented an anchor-based approach for multi-view semi-supervised, which constructs the affinity graphs by using an anchor-based strategy and obtains the optimal consensus graph by using feature and label information. Considering that original multi-view data often contain abundant noise and outliers, Xie et al. [63] learned latent feature representation based on the adaptively learned graph. It also introduces Laplacian embedding to maintain the local manifold structure. Zhang et al. [64] constructed a unified similarity matrix for multiple views by utilizing a latent representation explored from the underlying complementary information. Huang et al. [65] integrated similarity learning and local embedding into a unified framework, which constructs a fused similarity matrix and learns a latent low-dimensional representation for capturing the underlying structure. For preserving global structures and obtaining local structures, Wan et al. [66] proposed an embedding method for multi-view clustering, which integrates all views into a combination weight matrix for maintaining global structures and imposes constraint on the learned shared affinity matrix for obtaining the local structure.

## Proposed method

**Fig. 1:**
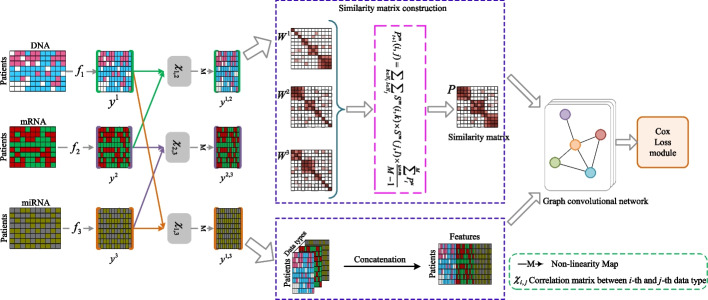
The architecture of our proposed MDJL

In this paper, we propose a Multi-type Data Joint Learning (MDJL) approach for cancer survival prediction based on multi-type genome-wide data. Specifically, instead of exploiting common feature information shared by all data types, we exploit correlation/common feature information between any two data types for exploring diverse and complementary feature information across multiple data types. Secondly, we fully utilize the global and local structure to construct similarity matrices based on the learned multiple correlation representations. Here, global structure refers to the similar structure information across different data types, local structure refers to the neighborhood information within data types. The main architecture of our MDJL approach is illustrated in Fig. 1. MDJL consists of four components: (1) correlation representations extraction component, which is designed for utilizing diverse and complementary feature information across multiple data types by learning correlation representations between any two data types; (2) discriminant representations generation component, which is designed for fusing multiple correlation representations by concatenation; (3) similarity matrices construction component, which is designed for generating sample similarity matrix by fully utilizing both global and local structure across different data types; and (4) graph convolutional network construction component, which is used for predicting the survival risk for patients. Key notations used in this paper are listed in Table 1.

**Table 1:** Summary of the key notations used in the paper

Notations	Explanations
${\textbf {x}}^{v}\in \mathbb {R}^{d^{v}\times N}$	Sample set of *v*-th data type
$x_{i}^{v}\in \mathbb {R}^{d^{v}}$	The *i*-th sample in *v*-th data type
$f_{v}$	Fully connection neural network used for feature learning
${\textbf {w}}_{f_{v}}^{l}\in \mathbb {R}^{m_{l}\times m_{l-1}}$	The *l*-th layer weight matrix of neural network $f_{v}$
${\textbf {y}}^{v}\in \mathbb {R}^{d\times N}$	The learned feature representation from ${\textbf {x}}^{v}$ with $f_{v}$
$\chi ^{v,u}\in \mathbb {R}^{d\times d\times N}$	Interactive map set between data type *v* and data type *u*
$\chi _{i}^{v,u}\in \mathbb {R}^{d\times d}$	Interactive map of *i*-th sample between data type *v* and data type *u*
${\textbf {y}}^{v,u}\in \mathbb {R}^{d\times N}$	The correlation representation of ${\textbf {x}}^{v}$ and ${\textbf {x}}^{u}$
${\textbf {y}}\in \mathbb {R}^{(V(V-1)/2)\times N}$	Fused correlation representation
$P^{m}$	Normalized weight matrix
$S^{m}$	K nearest similarity matrix
*P*	Fused similarity matrix
$z_{i}$	The output of graph convolutional network

### Correlation representations extraction

Suppose there are *N* samples and *V* different data types. Let ${\textbf {x}}^{v}=\left\{ x_{i}^{v}\in \mathbb {R}^{d_{v}} \right\} _{i=1}^{N}$ be the sample set of the *v*-th data type, and $x_{i}^{v}$ represents the *i*-th sample of data type *v*, $d_v$ is the feature dimensionality of ${\textbf {x}}^{v}$, where $v=1,2,\ldots ,V$. For correlation representation extraction, we firstly define *V* neural networks $\left\{ f_{v}\right\} _{v=1}^{V}$ to conduct feature learning and project ${\textbf {x}}^{v}$ from space $\mathbb {R}^{d_{v}}$ into space $\mathbb {R}^{d}$, that is,

1 $$\begin{aligned} {\textbf {y}}^{v} = f_{v}\left( {\textbf {x}}^{v}\right) , \end{aligned}$$

where ${\textbf {y}}^{v}\in \mathbb {R}^{d\times N}$, and $f_{v}$ points to a neural network with $L=3$ layers,

2 $$\begin{aligned} {\textbf {h}}_{f_{v}}^{l} =\sigma \left( {\textbf {w}}_{f_{v}}^{l}{} {\textbf {h}}_{f_{v}}^{l-1}+{\textbf {b}}_{{f_{v}}}^{l} \right) . \end{aligned}$$

For the *l*-th layer $\left( l = 1,2,\ldots ,L\right)$, ${\textbf {w}}_{f_{v}}^{l}\in \mathbb {R}^{m_{l}\times m_{l-1}}$ denotes the weight matrix $\left( m_{0} = d_{v}, m_{L}=d\right)$, ${\textbf {b}}_{f_{v}}^{l}\in \mathbb {R}^{m_{l}}$ is the bias vector, ${\textbf {h}}_{f_{v}}^{l}\in \mathbb {R}^{m_{l}}$ denotes the output of the *l*-th layer $\left( {\textbf {h}}_{f_{v}}^{0}={\textbf {x}}^{v}, {\textbf {h}}_{f_{v}}^{L}={\textbf {y}}^{v}\right)$, and $\sigma$ is the acivation function.

To further explore the correlation representations between any two data types, we borrow correlation computation proposed in [67]. Following work [67], for the *i*-th sample, the interactive map $\chi _{i}^{v,u}$ of $y_{i}^{v}$ and $y_{i}^{u}$ can be defined as,

3 $$\begin{aligned} \chi _{i}^{v,u} = y_{i}^{v} \otimes y_{i}^{u}, \end{aligned}$$

where $v\ne u$, $\otimes$ is outer product, $\chi ^{v,u}=\left\{ \chi _{i}^{v,u}\in \mathbb {R}^{d\times d} \right\} _{i=1}^{N}$, $\chi ^{v,u} = \chi ^{u,v}$.

Based on the interactive map set, we further construct a set of neural networks $\psi =\left\{ \psi _{v,u}\right\} _{v,u=\left\{ 1,\ldots ,V\right\} ,v\ne u}$ to project each $\chi ^{v,u}$ from space $\mathbb {R}^{d\times d}$ into an embedded space $\mathbb {R}^{d}$, which learns deep correlation representations between any two data types. That is,

4 $$\begin{aligned} {\textbf {y}}^{v,u}=\psi _{v,u}\left( \chi ^{v,u} \right) =\sigma \left( {\textbf {w}}_{\psi _{v,u}}\text {vec}\left( \chi ^{v,u} \right) +{\textbf {b}}_{\psi _{v,u}} \right) . \end{aligned}$$

where ${\textbf {y}}^{v,u}\in \mathbb {R}^{d\times N}$ is the correlation representation of ${\textbf {x}}^{v}$ and ${\textbf {x}}^{u}$, ${\textbf {w}}_{\psi _{v,u}}\in \mathbb {R}^{d\times d^{2}}$, ${\textbf {b}}_{\psi _{v,u}}\in \mathbb {R}^{d}$, $\text {vec}\left( \cdot \right)$ represents the vectorization of a matrix.

### Discriminant representations generation

Based on the above subsections, we have learned multiple correlation representations from multiple data types. The finally fused correlation feature representation from all pairwise data types can be written as,

5 $$\begin{aligned} \begin{aligned} {\textbf {y}}=&[{\textbf {y}}^{1,2\; \top },{\textbf {y}}^{1,3\; \top },\ldots ,{\textbf {y}}^{1,V\; \top }, \\&\ldots , \\&{\textbf {y}}^{v,v+1\; \top },{\textbf {y}}^{v,v+2\; \top },\ldots ,{\textbf {y}}^{v,V\; \top },\\&\ldots , \\&{\textbf {y}}^{V-1,V\; \top }]^{\top }. \end{aligned} \end{aligned}$$

### Similarity learning of global and local structure

As mentioned above, MDJL aims to learn a fused similarity matrix based on multi-type data. The reliability of the similarity matrices constructed from raw data may be polluted severely by noise and outliers. To enhance the ability to resist noise and outliers, we construct similarity matrices based on the learned multiple correlation representations. By correlation information learning, we collect *M* different correlation feature representations $\left\{ {\textbf {o}}^{m}={\textbf {y}}^{v,u}\in \mathbb {R}^{d\times N}\right\} _{m=1}^{M}$, where $M=V\left( V-1 \right) /2$. Based on the multiple correlation representations, similarity learning of global and local structure aims to capture a fused similarity matrix, which preserves sufficient local structure information of samples as well as maintains global structure across different data types. First, we construct the similarity matrix ${\textbf {W}}^{m}=\left[ W^{m}(i,j) \right] _{N\times N}$ for the *m*-th correlation representation ${\textbf {o}}^{m}$ by Gaussian kernel. $W^{m}(i,j)$ represents the similarity between sample $x_{i}^{m}$ and $x_{j}^{m}$ in the *m*-th correlation representation. To integrate these similarity matrices constructed from multiple correlation representations, we introduce a normalized weight matrix $P^{m}$ as follows:

6 $$\begin{aligned} P^{m}(i,j)=\left\{ \begin{array}{c} \frac{W^{m}(i,j)}{2\sum _{k\ne i}^{N}W^{m}(i,k)},j\ne i\\ 1/2,j=i \end{array}\right. \end{aligned}$$

where $$\sum _{j=1}^{N}P^{m}\left( i,j \right) =1$$.

In order to measure local similarity, we design a sparse kernel based on K nearest neighbors (KNN), that is:

7 $$\begin{aligned} S^{m}(i,j)=\left\{ \begin{array}{c} \frac{W^{m}(i,j)}{\sum _{k\in N_{i}^{m}}W^{m}(i,k)},j\in N_{i}^{m}\\ 0,otherwise \end{array}\right. \end{aligned}$$

where $N_{i}^{m}$ is a set of neighbors for $y_{j}^{m}$. This operation sets the similarities of samples that are non-neighboring to zero, which bases on pairwise samples similarity values.

To obtain fused similarity matrix, we iteratively update $P^{m}$ with its corresponding local similarity matrix $S^{m}$ and the similarity matrix $\left\{ P^{u}\right\} _{u=\left\{ 1,\ldots ,M \right\} \setminus m}$ of other data types, so that the updated $P^{m}|_{m=1}^{M}$ can be more similar to each other, at the same time, local similarity information can also be preserved.

For *m*-th correlation representation, we iteratively update $P^{m}$ as follows:

8 $$\begin{aligned} P_{t+1}^{m}(i,j)=\sum _{k\in N_{i}}\sum _{l\in N_{j}}S^{m}(i,k)\times S^{m}(j,l)\times \frac{\sum _{u\ne m}^{M}P_{t}^{u}}{M-1} \end{aligned}$$

After *T* iterations, the learned $P^{m}|_{m=1}^{M}$ would be enough similar to each other. Then the fused similarity matrix can be defined as the average of $P^{m}|_{m=1}^{M}$, that is:

9 $$\begin{aligned} P=\frac{\sum _{m=1}^{M}P_{T}^{m}}{M} \end{aligned}$$

### Graph convolutional network

According to correlation representations learning, we obtain the fused discriminant representation matrix ${\textbf {y}}$. According to similarity matrices construction, we obtain the fused similarity matrix *P*. Then the ${\textbf {y}}$ and *P* were used as the input of graph convolutional network for model training and prediction. In this paper, we construct the graph convolutional network $G = f({\textbf {y}},P)$ with three layers for training and prediction, that is,

10 $$\begin{aligned} H_{g}^{l+1}=\sigma \left( \tilde{D}^{-\frac{1}{2}}\tilde{P}\tilde{D}^{-\frac{1}{2}} H_{g}^{l}W_{g}^{l}\right) \end{aligned}$$

where $\tilde{P}=P+I_{N}$ denotes the adjacency matrix of the undirected graph *G* with added self-connections. $I_{N}$ represents identity matrix, $\tilde{D}_{(i,i)}= \sum _{j}\tilde{P}_{(i,j)}$, $W_{g}^{l}$ is trainable weight matrix of the *l*-th layer, $H_{g}^{l}$ points to the matrix of activations in the *l*-th layer ($H_{g}^{0}={\textbf {y}}$), and $\sigma$ is the activation function.

To describe the effectiveness of quantitative variables on survival time, we introduce Cox loss as loss function [25], that is,

11 $$\begin{aligned} L\left( \beta \right) =-\sum _{i:C(i)=1}\left[ \phi _{i} -log\left( \sum _{t_{j}\geqslant t_{i} } e^{\phi _{j} }\right) \right] \end{aligned}$$

12 $$\begin{aligned} \phi _{i}= z_{i}\beta \end{aligned}$$

where $\phi _{i}$ denotes the log hazard ratio for sample *i*, $z_{i}$ denotes the learned vector from graph convolutional network, $\beta$ represents coefficient weight vector between $z_{i}$ and the output $\phi _{i}$. *C*(*i*) is the censorship flag. If sample *i* is uncensored sample, $C(i)=1$, otherwise, if sample *i* is censored sample, $C(i)=0$. $t_{i}$ points to the survival time for patient *i*, where patient *i* should be uncensored samples. $t_{j}\geqslant t_{i}$ points to the survival time of *j*-th sample is longer than that of *i*-th sample, where patient *j* can comes from either uncensored samples or censored samples.

### Optimization

#### Feedforward and calculate the loss

For each of the *V* data types, the sample set ${\textbf {x}}^{v}$ are fed forward to the MDJL as in Eq. 1, and the output of the MDJL is denoted as $\left\{ z_{i} \right\} _{i=1}^{N}$. The loss of the whole network is calculated as in Eq. 11, denoted as $L\left( \beta \right) =-\sum _{i:C(i)=1}\left[ \phi _{i} -log\left( \sum _{t_{j}\geqslant t_{i} } e^{\phi _{j} }\right) \right]$.

#### Update neural networks

$\left\{ \left\{ f_{v} \right\} _{v=1}^{V},\left\{ \psi _{v,u}\right\} _{v,u=\left\{ 1,\ldots ,V\right\} ,v\ne u},G \right\}$. The network parameters of $\left\{ \left\{ f_{v} \right\} _{v=1}^{V},\left\{ \psi _{v,u}\right\} _{v,u=\left\{ 1,\ldots ,V\right\} ,v\ne u},G \right\}$ can be jonintly optimized by minimizing Eq. 11. We perform batch gradient descent with the whole dataset in each iteration for network training.

**Table:** 

**Algorithm 1** Algorithm for MDJL	
**Input**: sample set $\left\{ {\textbf {x}}^{v}\in \mathbb {R}^{d_{v}\times N}\right\} _{v=1}^{V}$, sample survival time set, sample survival status set.	
**Initialize**: hyperparameters *K*, *T*.	
**Update until convergence**:	
**Forward propagation**:	
1. Perform $f_{v}$ with Eq.1 and then obtain ${\textbf {y}}^{v}$.	
2. Compute interactive map $\chi ^{v,u}$ with Eq.3.	
3. Obtain correlation representations ${\textbf {y}}^{v,u}$ with Eq.4.	
4. Obtain fused correlation representations with Eq.5.	
5. Construct normalized weight matrix $P^{m}|_{m=1}^{M}$ with Eq.6.	
6. Construct sparse kernel matrix $S^{m}|_{m=1}^{M}$ with Eq.7.	
7. Iteratively update $P^{m}|_{m=1}^{M}$ with Eq.8.	
8. Obtain fused similarity matrix *P* with Eq.9.	
9. Construct gaph convolutional network *G* with Eq.10.	
**Back propagation**:	
Update network parameters of $\left\{ f_{v}\right\} _{v=1}^{V}$, $\left\{ \psi ^{v,u} \right\} _{v,u=\left\{ 1,2,\ldots ,V\right\} ,v\ne u}$	
and *G* by minimizing Eq.11.	
**Output**: The predicted hazard ratios of testing samples.	

**Algorithm 1** describes the process of cancer survival prediction by using MDJL.

## Experiments

### Datasets

**Fig. 2:**
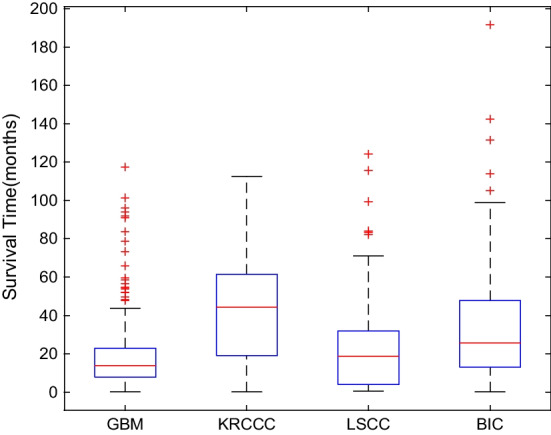
Survival time distribution of patients for four cancers

Four cancer datasets^1^ including glioblastoma multiforme (GBM), kidney renal clear cell carcinoma (KRCCC), lung squamous cell carcinoma (LSCC) and breast invasive carcinoma (BIC) are used to evaluate our MDJL approach. For each dataset, we collect three types of genomic data, including DNA methylation, mRNA expression and miRNA expression data. The datasets used in this paper are obtained from http://compbio.cs.toronto.edu/SNF/, which are provided and preprocessed by work [68]. It downloads these data from The Cancer Genome Atlas (TCGA) website and performs three steps of preprocessing: sample selection, missing-data imputation and normalization. Detailed preprocessing process is described as follows: (i) if one patient sample has more than 20% missing data in any data type, then this sample will be removed; (ii) if a certain gene has more than 20% missing values, then this gene will be filtered, otherwise, the k-nearest interpolation is used for complementing this gene; (iii) the z-score transformation is used for normalizing the data samples. Table 2 summaries the detailed information of datasets used in experiments. Figure 2 describes the survival time distribution for each cancer, which is represented by box plot.

**Table 2:** A brief statistics of all datasets

Cancer type	GBM	KRCCC	LSCC	BIC
Instances	215	122	106	105
Uncensored	199	79	66	83
Description of data types	DNA (12042-D)	DNA (17899-D)	DNA (12042-D)	DNA (17814-D)
	mRNA (534-D)	mRNA (329-D)	mRNA (352-D)	mRNA (354-D)
	miRNA (1305-D)	miRNA (24960-D)	miRNA (23074-D)	miRNA (23094-D)
Average survival time (months)	19.6	42.8	24.3	34.8

### Experimental settings

#### Compared methods

To evaluate the performance of our MDJL approach, we compare it with several state-of-the-art cancer survival prediction methods:

- MKL + Cox loss (MKL-Cox). MKL is a multiple kernel learning based binary classification method for cancer survival prediction, which fuses multi-type data using joint strategy [21]. For a fair comparison, we extend MKL with Cox loss.
- MDNNMD + Cox loss (MDNNMD-Cox). MDNNMD is a multimodal deep neural network based binary classification method for cancer survival prediction, which fuses multi-type data using joint strategy [37]. For a fair comparison, we extend MDNNMD with Cox loss.
- DLMR. DLMR is a multimodal deep neural network extension of the Cox model for cancer survival prediction, which fuses multi-type data using alignment strategy [54].
- CrossAE. CrossAE is a cross-modality autoencoder based survival prediction method for utilizing the consensus representations across multi-type data [53].
- VAECox. VAECox is a deep transfer learning architecture for cancer survival prediction based on alignment strategy [25].
- DeepSurv. DeepSurv is a deep learning generalization of the Cox proportional hazards model, which predicts survival risks based on single-type data [51]. For comparison, we use the unified feature matrix concatenated from DNA, mRAN and miRAN as the input for DeepSurv.

The implementations of MDNNMD-Cox, DLMR, CrossAE, VAECox and DeepSurv are downloaded from the websites provided by their authors. With there are no public codes for MKL-Cox, we implement MKL-Cox by ourselves.

#### Implementation details

All these methods are evaluated on GBM, KRCCC, LSCC and BIC datasets. For each cancer dataset, we randomly select 70% data for training and utilize the rest of 30% for testing. The details of network architecture for MDJL are as follows: For feature learning, we design the networks $\left\{ f_{v}\right\} _{v=1}^{V}$ with second and third layer of size 512 and 128. For prediction, we construct a three-layer graph convolutional network with hidden layer containing 32 nodes. For the network architecture, we adopt Adam optimizer and set the learning rate as 0.0001. In addition, we set hyper-parameters *K*=20, and *T*=30 in similarity matrix fusion algorithm. In this paper, the concordance index (C-index) is adopted to evaluate the performance of the competing survival prediction models, which mainly measures the proportion of all sample pairs for which the predictions and actual results are consistent. In order to guarantee fairness and robustness of research methods, for each dataset, we conduct 20 trials for each compared method, and the average performance of 20 trials is reported. For each trial, we would re-split the training and testing sets with 70% data for training and 30% data for testing, and re-fit the models. The corresponding Python code for carrying out our method is available at https://github.com/githyr/MDJL_Survival.

### Experimental results

**Fig. 3:**
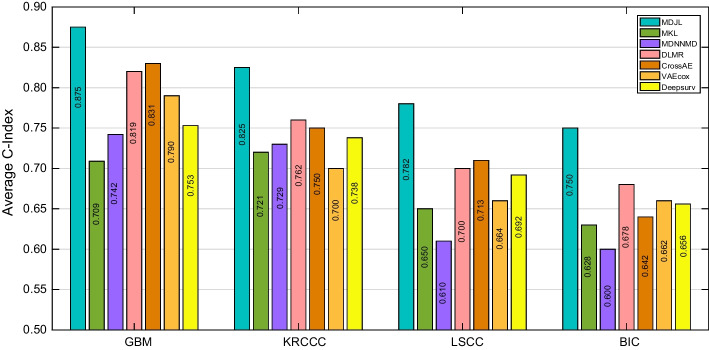
The performance of our approach and compared methods on all the datasets

The predictive results of all competing methods are reported in Fig. 3, from which we can observe that our MDJL approach outperforms other competing methods on four cancer types in terms of average concordance index (C-index). In general, compared with the second best method, our approach improves the average prediction performance by 4.40%, 6.30%, 6.90% and 7.2% on the GBM, KRCCC, LSCC and BIC datasets, respectively. The reasons are two-fold: Firstly, our approach exploits correlation information between any two data types, which can learn more useful information as well as reduce noise more thoroughly than joint based and alignment based methods. In addition, we further explore structural information, which can help learn effective feature representations with small sample size.

**Fig. 4:**
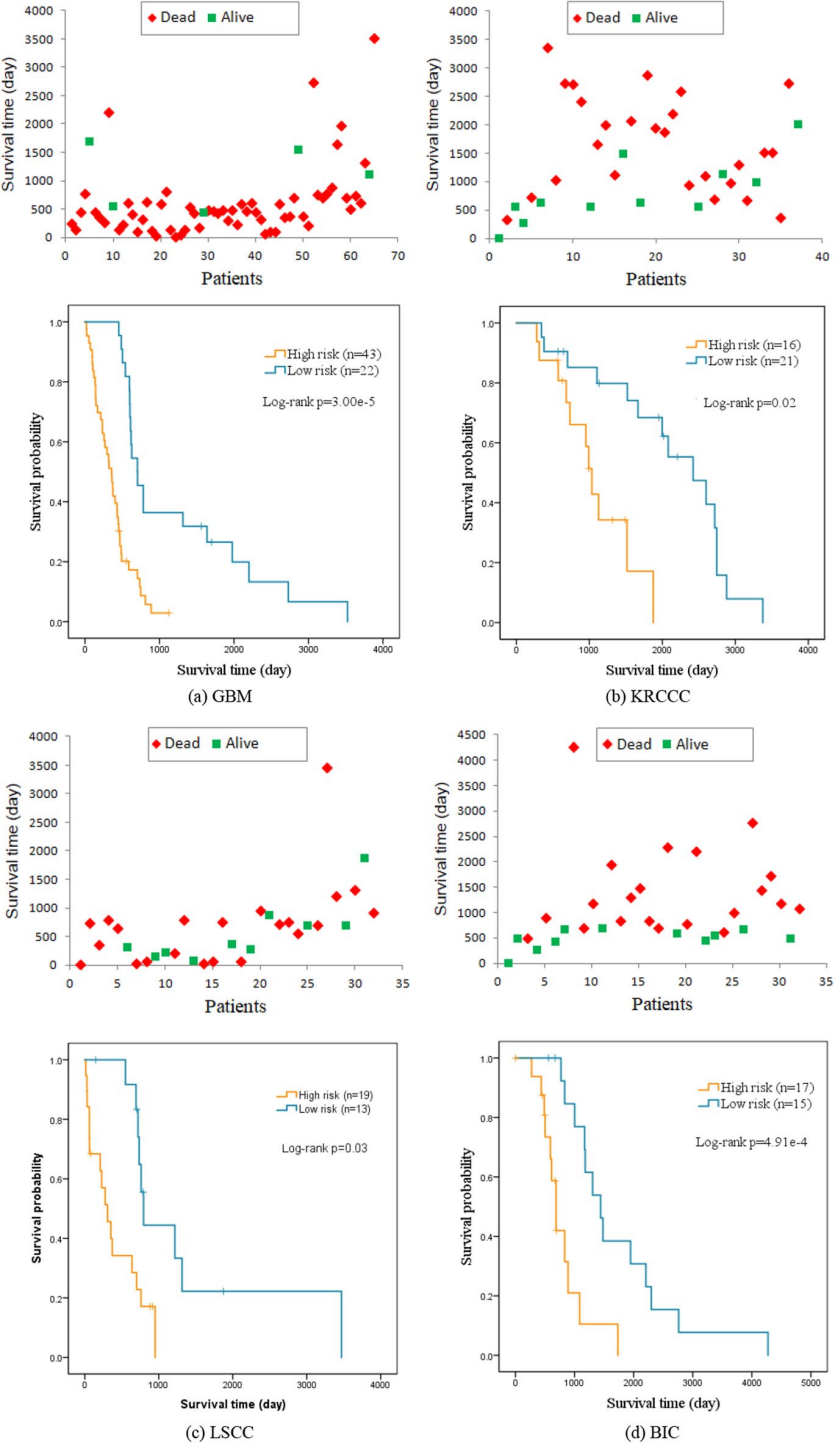
Survival analysis of test sets for four cancers. The top plots in **a**–**d** present the survival time of test sets samples with the red dots pointing to dead patients and the green dots pointing to censored patients. The bottom plots in **a**–**d** show the KM survival curves

We further investigate our MDJL approach with survival analysis which can be regarded as a statistical method considering both results and survival time. The patient samples for each cancer type would be divided into high-risk and low-risk groups based on their predicted hazard ratios. For example, a patient sample would be assigned to high-risk group if his hazard ratio is higher than the median hazard ratios of all patient samples, otherwise, he would be included in low-risk group. We illustrate the Kaplan-Meier (KM) curves in Fig. 4, which can reflect the survival condition of a group. The survival curve is a broken line, with each step corresponding to a time point of death and each mark pointing to a sample censoring, and P values are computed according to the curves. From the figure, we can observe that the survival probability of each group gradually drops with the increase of survival time, and the P-values for GBM, KRCCC, LSCC and BIC are $3.00\times 10^{-5}$, 0.02, 0.03 and $4.91\times 10^{-4}$, respectively, which are all smaller than 0.05. From the KM curves and the P-values, we can conclude that our approach can achieve a convinced result for predicting the high-risk or low-risk of one patient sample.

## Further investigation

### Effectiveness of correlation representation extraction

In this section, we verify the effectiveness of correlation representation extraction. In this paper, we integrate multiple data types for exploiting discriminant features by exploiting correlation information between any two data types, instead of exploiting common information shared by all data types or directly concatenating original multiple data types. In this paper, we call the version of exploiting common information shared by all data types for learning discriminant feature representations as CIAD, and the version of directly concatenating original multiple data types for learning discriminant feature representations as COMD. For CIAD, we exploit shared feature matrix by constructing feature learning networks for each data type and imposing Euclidean distance constraint between the learned feature representations of any two data types, and construct similarity matrices based on original multiple data types. For COMD, we concatenate original multiple data types into a unified feature matrix, and construct similarity matrices based on original multiple data types.

**Fig. 5:**
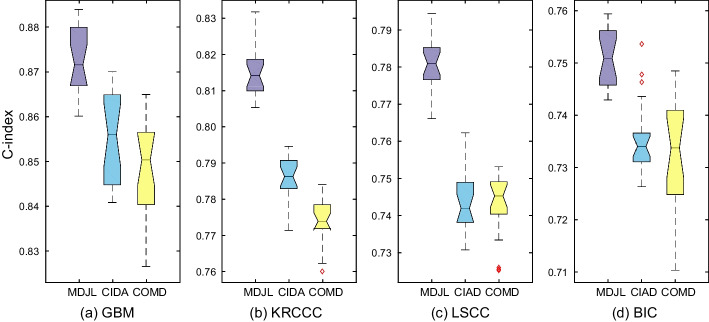
The box plot for the performance of different discriminant feature learning strategies

We perform MDJL, CIAD and COMD on each cancer dataset respectively for 20 trials and record the C-index score for each performance. For each trial, we would re-split the training and testing sets with 70% data for training and 30% data for testing, and re-fit the models. Figure 5 illustrates the C-index for 20 times with box plot. From the figure, we can observe that our approach outperforms the other two versions on four cancer types. As a summary, learning discriminant feature representations by exploiting correlation information between any two data types can achieve better performance than exploiting common information shared by all data types or directly concatenating original multiple data types.

### Effectiveness of learning structure information

In this section, we verify the effectiveness of learning structure information based on correlation representations. We respectively perform the model with learning structure information based on correlation representations, the model with learning structure information based on original data, and the model without learning structure information. We call the version that utilizes original multi-type data to construct similarity matrices as MDJL-OS, and call the version of MDJL without learning structure information as MDJL-SI. For MDJL-OS, we utilize original multi-type data to construct similarity matrices and exploit discriminant feature representations by learning correlation information between any two data types. For MDJL-SI, we exploit discriminant feature representations by learning correlation information between any two data types and replace the graph convolutional network with a three-layer fully connected network.

**Fig. 6:**
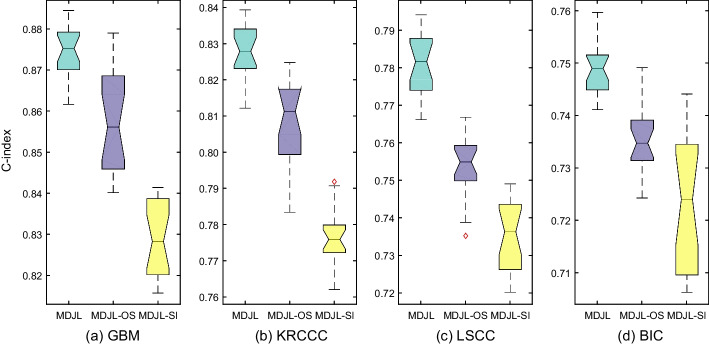
The box plot for the effectiveness of learning structure information (C-index)

We perform MDJL, MDJL-OS and MDJL-SI on each cancer dataset respectively for 20 trials and record the C-index score for each performance. For each trial, we would re-split the training and testing sets with 70% data for training and 30% data for testing, and re-fit the models. Figure 6 reports the C-index scores for 20 times with box plot, from which we can see that: (1) the performance for MDJL is better than that for MDJL-OS and MDJL-SI; (2) the performance for MDJL-OS is better than that for MDJL-SI. These results in this figure confirm that: (1) compared with only utilizing feature information, joint learning structure information and feature information can achieve better performance; (2) compared with constructing similarity matrices with original data, constructing similarity matrices with the learned correlation features can achieve better performance.

**Fig. 7:**
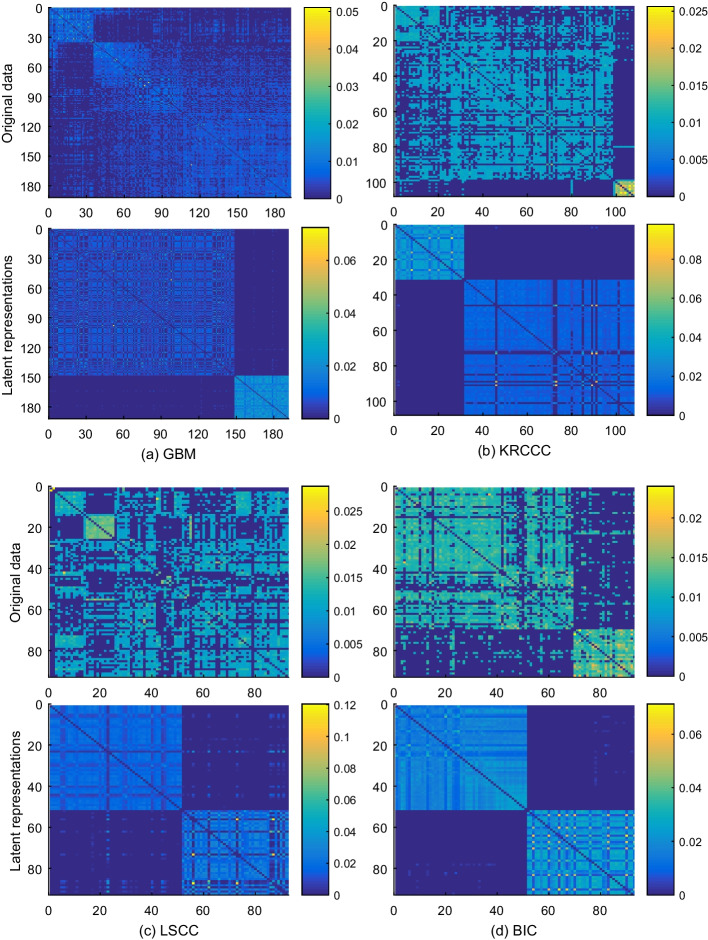
Experimental visualizations of the fused similarity matrices on four cancer datasets. The top plots in **a**–**d** present the fused similarity matrices learned from original data. The bottom plots in **a**–**d** illustrate the fused similarity matrices learned from correlation information

To further investigate the effective of the fused similarity matrices respectively learned from multiple correlation representations, we exhibit the fused similarity matrices of the training sets on four cancer datasets in Fig. 7. From the figure, we can observe that the outline of the similarity matrices learned from multiple correlation representations are obvious than these learned from original multiple data types on all four cancer datasets. The reason is that the original data is unfavorable to the estimation of similarity matrices.

### Parameter analysis

**Fig. 8:**
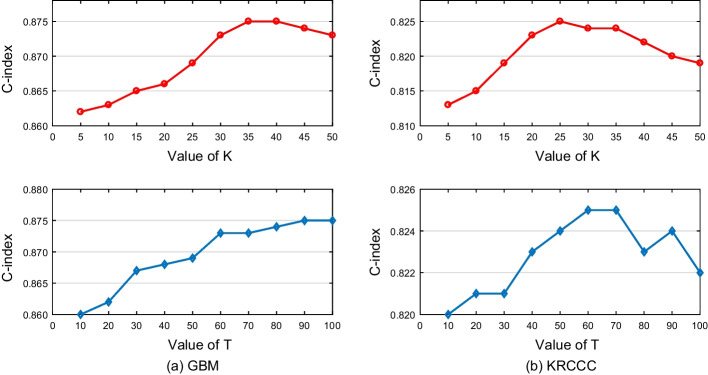
The performance of our approach with different values of *K* or *T* on GBM and KRCCC

In this section, we investigate the sensitivity for hyper-parameters *K* and *T* with fixing any one hyper-parameter and changing the value of another hyper-parameter. When *K* is evaluated, we set *T* as 50. When *T* is evaluated, we set *K* as 20. We repeat each execution 20 times and record the average C-index. For each trial, we would re-split the training and testing sets with 70% data for training and 30% data for testing, and re-fit the models. Figure 8 shows the C-index of our MDJL approach versus different values of *K* and *T* on GBM and KRCCC. From the figure, we can observe that the C-index of MDJL on GBM and KRCCC datasets have a small fluctuation range (< 0.2). In general, the proposed approach is insensitive to hyper-parameters *K* ranging from 5 to 50 and *T* ranging from 10 to 100.

### Computing time

In this section, we use the model training time iterating over all the datasets 200 times to measure the computing time of MDJL and other baselines. Computing time of all compared methods is collected from a computer with an Intel i7 quadcore 3.6GHz CPU, a NVIDIA GTX1080Ti GPU, and 16GB memory. As seen from Table 3, the computing time of MDJL is acceptable.

**Table 3:** Comparison of training time (Seconds)

Datasets	Methods						
	MDJL	MKL	MDNNMD	DLMR	CrossAE	VAEcox	DeepSurv
GBM	81.57	59.74	72.86	63.85	86.23	52.76	41.52
KRCCC	118.31	90.44	103.81	96.28	130.84	79.56	64.23
LSCC	71.26	54.17	62.92	57.36	80.17	48.57	36.76
BIC	116.45	84.23	95.34	90.04	130.81	73.58	59.64

## Conclusion

In this paper, we propose a novel multi-type data joint learning approach, and apply it to the cancer survival prediction task. MDJL integrates correlation representation learning, similarity learning and graph convolutional network construction into a unified framework. Correlation feature representations between any two data types are effectively and fully exploited to learn discriminant feature representations. Global and local structure information among samples is fully exploited to learn the relationships among samples.

Extensive experiments on four public cancer datasets demonstrate that our approach can achieve better performance than other competing cancer survival prediction methods. In addition, experiments also demonstrate the effectiveness of the designed modules of our approach.

## Data Availability

The datasets generated and analysed during the current study are available with http://compbio.cs.toronto.edu/SNF/.

## Footnotes

1 http://compbio.cs.toronto.edu/SNF/
